# Table olives and health: a review

**DOI:** 10.1017/jns.2020.50

**Published:** 2020-12-02

**Authors:** Janete Rocha, Nuno Borges, Olívia Pinho

**Affiliations:** 1 Faculdade de Ciências da Nutrição e Alimentação, Universidade do Porto, Porto, Portugal; 2 CINTESIS – Centro de Investigação em Tecnologia e Serviços de Saúde, Porto, Portugal

**Keywords:** Table olives, Mediterranean Diet, Monounsaturated fat, Alpha-tocopherol, Phenolic compounds, ALS, amyotrophic lateral sclerosis, AO, antioxidant, BP, blood pressure, cv, cultivar, CVD, cardiovascular disease, DM-II, Diabetes Mellitus 2, e.p, edible portion, EFSA, European Food Safety Authority, FM, fat mass, GSH, glutathione, HDL-c, high-density lipoprotein cholesterol, HT, hydroxytyrosol, LDL-c, low-density lipoprotein cholesterol, MD, Mediterranean Diet, MUFA, monounsaturated fat, NaCl, sodium chloride, NaOH, sodium hydroxide, NO, nitric oxide, Nrf2, nuclear factor erythroid 2-related factor 2, OL, oleuropein, OO, olive oil, PKC, protein kinase C, PUFA, polyunsaturated fat, RDA, Recommended Dietary Allowance, ROS, reactive oxygen species, TC, total cholesterol, TG, triacylglycerol, TG, triglyceride, Ty, tyrosol, WHO, World Health Organization, α-TOH, alpha-tocopherol

## Abstract

Table olives, a product of olive tree (*Olea europaea* L.), is an important fermented product of the Mediterranean Diet. Agronomical factors, particularly the cultivar, the ripening stage and the processing method employed are the main factors influencing the nutritional and non-nutritional composition of table olives and their organoleptic properties. The important nutritional value of this product is due to its richness in monounsaturated fat (MUFA), mainly oleic acid, fibre and vitamin E together with the presence of several phytochemicals. Among these, hydroxytyrosol (HT) is the major phenolic compound present in all types of table olives. There is a scarcity of *in vitro*, *in vivo* and human studies of table olives. This review focused comprehensively on the nutrients and bioactive compound content as well as the health benefits assigned to table olives. The possible health benefits associated with their consumption are thought to be primarily related to effects of MUFA on cardiovascular health, the antioxidant (AO) capacity of vitamin E and its role in protecting the body from oxidative damage and the anti-inflammatory and AO activities of HT. The influence of multiple factors on composition of the end product and the potential innovation in the production of table olives through the reduction of its final salt content was also discussed.

## Introduction

*Olea europaea* L., commonly known as the olive tree, is native in the Mediterranean region and is one of the oldest tree species whose fruit, also called olive, and by-products, such as table olives and olive oil (OO), have historically served as the basis of the nourishment for indigenous populations in this region^(1)^. Table olives are a food product obtained from the processing of the olive fruit. Table olives are important fermented vegetables of the Mediterranean Diet (MD), and in agreement with MD pyramid guidelines, olives, nuts and seeds should be consumed every day in a moderate amount of 1–2 portions (such as a handful), representing a healthy snack option^(2)^.

In spite of their common origin, table olives and OO have been analysed and studied in a different manner, and a large number of studies that support the current scientific evidence about the health effects associated with consumption of OO (particularly extra-virgin OO) can be found. In contrast, it is possible to verify the scarcity of studies in table olives, namely *in vitro*, *in vivo* and human studies.

The aim of the present review was to compile the available evidence related to composition and health effects expected from consumption of table olives and the factors influencing their nutritional, non-nutritional and organoleptic characteristics.

The search procedures for studies and reviews on these topics were performed using the PubMed and Scopus databases between January and May 2019, unlimited in the time period of publication.

## Table olive production and consumption worldwide

According to the report of the International Olive Council, the worldwide production of table olives has increased systematically since the 1990–1 season until the 2016–17 season from 950 000 to 2 889 000 tones. The majority of the production is located in the European Union (EU), particularly from Mediterranean countries (Spain, Greece, Italy and Portugal). Other significant producing countries include Egypt, Turkey, Syria and Morocco^(3,4)^.

The worldwide consumption of table olives has also increased since the 1990–1 season, although in a milder way, and amounted to 2 724 000 tones, where Egypt, Turkey and Algeria were the major consumers^(5)^. In the 2016–17 season, the EU consumption was 572 000 tones, being Spain the main consumer^(6)^.

## Morphological and chemical composition of olives

The olive fruit is a drupe, single-seeded with a fleshy outer layer that structurally can be separated into three distinct anatomical parts such as epicarp (skin), mesocarp (pulp or flesh) and endocarp (kernel), containing the seed^(7,8)^. The epicarp is a protective tissue representing 1⋅0–3⋅0 % of the drupe weight. The colour of epicarp changes with its maturation, going from bright green in early stages of development, due to the accumulation of chlorophyll to pale green, straw yellow, pink, purple pink and black. The marked colour changes which occur are caused by unbalanced and varying concentrations of the major pigments in olives such as chlorophylls, carotenoids and anthocyanins^(8)^. The epicarp and the mesocarp form the edible portion (e.p.) of olive fruit and represent around 70–85 % of the olive weight. The mesocarp is the reserve tissue of all constituents, namely water (70–75 % of the mesocarp weight) and fat (ranging from 14 to 15 % in green table olives and about 30 % in black mature olives)^(8,9)^. The endocarp is characteristic of a variety, representing 18–22 % of the olive weight. The kernels comprise 2–4 % of the weight of endocarp and contain a considerable amount of fat (about 22–27 %)^(8)^. It is important to note that all three parts influence the quality of the end product^(9)^.

The nutritional and non-nutritional composition of table olives is determined qualitatively and quantitatively by agronomical factors, the ripening stage of the fruit and the method of processing used. These factors also determine the organoleptic properties of the final product, such as taste, flavour, colour and texture, thus influencing the commercial value of table olives^(7,10)^.

### Agronomical factors

The composition of table olives varies with cultivar (cv.), agricultural practices, irrigation management, agroclimatic conditions and geographical origin^(7,11)^. Among these factors, cv. is the one with the biggest influence. Cvs. vary considerably in size, shape, fat content and flavour^(7)^. Phenolic composition of table olives is mainly driven by genetic factors and significant differences exist between different olive cvs.^(7,12)^.

### Ripening stage

The moment of harvesting the fruit, and its correspondent ripening stage, depends on the processing method and the style of table olives. However, most olives are harvested in mid-autumn when they are firm and the colour changes from green to yellowish green^(13,14)^.

As the maturation of olives progressively occurs, the fat content of the olive flesh increases simultaneously with a decrease of water content; therefore, green olives have lower fat content compared with black olives^(8,14)^. The phenolic compounds also change qualitatively and quantitatively with the ripening stage of olive fruit, along with a change in the pigment levels. During the growth phase of olive fruit, the total phenol concentration, in particular oleuropein (OL; a secoiridoid glucoside), increases to a maximum level at the green maturation phase. Thereafter, a noticeable change in the phenolic fraction occurs, with a reduction in total phenolic compounds, especially OL, although there is a continuous synthesis of these compounds until maturity^(7,13)^. Ligstroside also decreases as the fruit develops^(8)^. In the meantime, an increase occurs in the hydroxytyrosol (HT), tyrosol (Ty), verbascoside, demethyloleuropein, elenolic glycoside and glucoside forms of flavonoids such as luteolin-7-O-glucoside, cyanidin-3-O-glucoside, cyanidin-3-O-rutinoside and quercetin-3-O-rutinoside concentrations^(12,13,15)^. Regarding the pigment levels, there are three distinguishable stages: green olives when chlorophyll and carotenoid levels are higher; turning colour olives when a partial decrease in chlorophyll levels occurs and black olives, where monomeric anthocyanins appear, mainly cyanidin-3-O-rutinoside and cyanind-3-O-glucoside, and chlorophyll and carotenoids no longer influence the colour of the olive fruit^(7,12,16,17)^.

### Processing method

Olives present a natural bitterness, mainly caused by secoiridoid OL, that should be removed to improve palatability of table olives thus increasing its acceptance by consumers^(18)^. Therefore, olive fruits must undergo a processing treatment to become edible for consumers. There are three important internationally used practices for the preparation of table olives: Spanish-style green olives, Californian-style black olives in brine (ripe olives can be picked in the green or turning colour stage) and Greek-style natural black olives in brine (directly brined olives)^(10,13)^. Nevertheless, other industrial techniques can be carried out. The choice of processing method is done according to the region and depends on the variety of olives^(13)^.

#### Spanish-style green olives

Olive fruits are harvested with colours varying from green to yellow but having reached normal size. Olives are submitted to a sodium hydroxide (NaOH) solution (lye, normally 1⋅3–2⋅6 % w/v) until NaOH reaches from two-thirds to three-fourths of the distance between the surface of the olives and the stone^(15)^. The concentration of NaOH used will depend on the temperature, cv. and degree of the fruit maturity. The lye treatment hydrolyses the OL into non-bitter HT and oleoside-11-methyl ester. Subsequently, olives are washed with water to remove the excess of lye and submitted to a sodium chloride (NaCl) solution (6–8 % w/v) for a mild lactic fermentation^(15,19)^. The levels of OL and other phenolics present in the brine can influence the fermentation rate, as they have antimicrobial activity. Finally, the olives are packed in brine (≥8 % w/v NaCl) and they can be further processed to prolong shelf-life, through the addition of sorbic acid or its salts, or submitted to pasteurization (62⋅4°C for 15 min)^(15)^.

#### Californian-style black olives in brine

Olive fruits are harvested before they reach their final maturation stage. First, olives are stored in brine (5–10 % w/v NaCl) for a period which varies from 2 to 6 months, with medium acidification until pH 4 through the addition of lactic and acetic acids and in anaerobic/aerobic conditions to prevent fermentation^(10,15)^. Posteriorly, fruits undergo a treatment with two to five NaOH solutions (1–2 % w/v), leading to a progressive entry of NaOH into the flesh^(15,20)^. In intervals between lye treatments, olives are suspended in water or a weak brine solution in which air is bubbled, leading to oxidation by aeration and polymerization of phenolic compounds, transforming them into different dark compounds, allowing that a rapid darkening of the fruit occurs^(12,15)^. Iron salts, such as ferrous gluconate or ferrous lactate, can be used to stabilize and maintain the black colour of table olives. The change in colour of olives is also facilitated by the formation of uncoloured ferrous complexes and the following oxidation to dark ferric iron complexes^(12)^. Normally after, these table olives are canned in brine and submitted to a sterilization treatment^(15)^.

#### Greek-style natural black olives

Olive fruits, when intended to be processed with this method, are harvested in the final stage of maturation with a dark colour. After harvesting, olives are washed and directly immersed in a brine solution (8–10 % w/v NaCl), without any debittering treatment^(12,15)^. A natural and spontaneous fermentation process starts, driven mainly by yeasts, due to high salt concentration used, and also by lactic acid and gram-negative bacteria. It is noteworthy that fermentation may be carried out in either anaerobic or aerobic conditions. The microbiota is defined by substrate availability, salt level, temperature and pH values, aerobic and anaerobic conditions and antimicrobial compounds present such as phenolic compounds. During fermentation, bitterness of olives is lost because of the diffusion of OL from the fruit to the brine and the posterior acid hydrolysis of this compound^(11,12,15,19)^.

#### Effects of processing olive fruits

Bearing in mind the production of table olives, the fruit's processing determines a change in its composition, particularly in the components present in less quantity such as phenolic compounds, and its organoleptic characteristics. The use of NaOH solutions is the most effective method in order to reduce the olive bitterness; however, it also determines greater losses, since NaOH solution sets larger differences in comparison to the losses that yeasting processes result in^(18)^.

The NaOH solutions induce an increase in the permeability of the epicarp, thus determining an increase in the rate of the OL hydrolysis and diffusion into the surrounding medium. The lye induces the hydrolytic cleavage of the ester bond on OL between HT and oleoside-11-methyl ester (elenolic acid glucoside). Verbascoside is also hydrolyzed through the same mechanisms, producing HT and caffeic acid. Ligstroside is hydrolyzed by producing Ty and the oleoside-11-methyl ester. This step also induces a decrease in rutin and luteolin-7-glucoside levels due to the hydrolysis of the glycosides^(12)^. Furthermore, NaOH and compounds with carboxylic and hydroxyl functional groups, such as carboxylic acids, polycyclic triterpenols, polycyclic terpene acids and glycosides, react and the hydrophilic derivatives are washed away^(8,12)^. These new formed compounds can diffuse into the brine and the rinsing water. The maslinic and oleanolic acid concentrations are also reduced in alkaline-treated olives, along with a decrease in alpha-tocopherol (α-TOH) driven by the use of lye^(12,18)^. It can be said that the alkaline treatment used in the Spanish-style and Californian-style methods results in table olives with lower levels of total phenols, such as HT and Ty, which strongly modify the flesh and epicarp structure, while having no influence on the triacylglycerol (TG) levels^(12,14,18)^.

The various steps involved in the Spanish-style method, particularly the lye, washing and brining of olives, cause the loss of the majority of the water-soluble compounds from the olive flesh. Additionally, the fermentation and the storage also induce modifications in the olive's composition by using nutrients and producing new compounds^(14)^.

In the Californian-style method, the oxidation by aeration causes a great decrease in phenolic compounds, such as HT and anthocyanins. The black ripe olives have null or lower concentrations of HT compared with table olives produced by the other methods^(12)^. As a matter of fact, the Californian-style contain much lower concentrations of total phenolic compounds compared with the Spanish- or Greek-style table olives, resulting in loss of nutritional value^(9,10,12,18)^. Moreover, the oxidation step induces a remarkable change in the texture of the flesh, promoting softening which decreases the commercial value of the end product^(8)^.

In the Spanish- and Greek-style method, a fermentation process occurs and its effect on phenol content of the table olives depends on the maturity stage of the olive fruits, the NaCl concentration, the duration of treatment, the temperature of solution and the different debittering methods previously used^(12)^. The osmotic pressure induced by NaCl used in brine curing also influences the diffusion of phenol compounds. Simultaneously, NaCl reaches olive tissue conferring the salty taste of the table olives^(12)^. During fermentation, the decrease of pH caused by the production of organic acids can favour the diffusion of HT, Ty and oleoside-11-methyl ester into the soaking medium until an equilibrium is reached. In contrast, aerobic microorganisms can prevent further oxidation of HT. In the production of naturally black table olives, an acid hydrolysis of HT, Ty and luteolin glycosides occurs during the fermentation^(12)^. It was observed that both spontaneous and controlled fermentations lead to loss of total phenol compounds of about 32–58 %, mainly driven by diffusion of these compounds into the brine, being HT the main phenolic compound identified and quantified in different brines^(13)^. The fermentation step also contributes to the sweetening of the olive fruits, but due to processing variability, the final table olives produced vary in colour, form and other sensorial parameters^(12,14)^.

Regarding the Greek-style method, part of the monomeric anthocyanins diffuses into the brine, although most polymerizes within the drupe into stable pigments, favouring the colour of the table olives^(17)^. However, the anaerobic fermentation process can induce loss of anthocyanins^(10)^. Contrary to Spanish- and Californian-style, the Greek-style does not influence the triterpenic acid levels, mainly maslinic and oleanolic acids, in table olives^(17,18)^. The natural maturation process of black olives is accompanied with the degradation of pectins and a consequent modification in texture^(8)^.

Further processing of table olives, particularly destoning, stuffing and seasoning, have a detrimental effect on the quality of the end products. Destoning table olives increase the surface area through which polyphenols can diffuse into the surrounding medium^(12)^. A flotation step is used to separate the pitted product from the non-pitted and the fragments of pulp, determining new dilutions of phenolic compounds in the washing liquids^(12)^.

According to the previously mentioned, regardless of the processing method used, noticeable chemical and physical change occurs, influencing the lipid constituents, sugars and salts and the texture of the fruit^(8,9)^, while TG and protein content does not change with such processing methods. A significant loss of total phenols occurs during the several steps, especially in the lye treatment and fermentation, associated with a decrease in the antioxidant (AO) capacity compared with fresh olives^(12)^.

## Nutritional characteristics of table olives

The energy content of 100 g e.p. of table olives ranges from 180 to 250 kcal with some exceptions (455 kcal/100 g e.p. for Majatica cv. and 164 kcal for Bella di Cerignola cv.)^(9)^. Beside moisture, which ranges from 60 to 81 g/100 g e.p. in different table olives^(14)^, lipids are the major component of table olives with an overall range of 6–30 g/100 g e.p.^(14,18)^. The protein level is low, ranging from 1⋅0 to 2⋅2 g/100 g e.p., and it does not vary much with table olive style. Despite its little contribution to overall nutritional value of table olives, the proteins present in table olives are of high quality as a result of the presence of all essential amino acids, though aspartic and glutamic acids being the most representative ones^(9,14)^. Carbohydrates are practically absent in table olives due to their transformation/removal during the fermentation/storage in brine^(14)^.

Lipids are the macronutrient with the most important role in the nutritional value of table olives, and there are clear differences within cvs. and processing styles^(14)^. The range of lipid concentration in green olives is rather wide varying from 6 to 24 g/100 g e.p. due to the diverse fat concentrations in the cvs. devoted to this style and in the stuffing materials^(14)^. Ripe olives also have a wide range of lipid content from 8 to 24 g/100 g e.p.^(14)^, and in directly brined olives, the lipids are in higher amount ranging from 18 to 28 g/100 g e.p.^(14)^ (Table 1). The lipidic fraction of table olives is mainly composed of TGs and contains small quantities of sterols, total fatty alcohols and triterpenic alcohols. The highest concentration of TGs was found in directly brined olives from 20 to 30 g/100 g e.p. depending on the cv., and green and ripe olives have similar concentrations ranging from 11 to 20 g/100 g e.p.^(14)^. The primary lipid biochemical profile of table olives is characterized by a high proportion of unsaturated and a low proportion of saturated fats. As shown in Table 1, table olives lipids consist of 66⋅8–82⋅1 %^(9)^ monounsaturated fat (MUFA) including 5⋅5–36⋅0 g/100 g e.p. oleic acid (C18:1 ω-9), 4⋅9–14⋅2 %^(9)^ polyunsaturated fat (PUFA) including 0⋅7–3⋅75 g/100 g e.p. linoleic acid (C18:2 n-6) and less than 22⋅4 %^(9)^ saturated fats including 1⋅2–5⋅1 g/100 g e.p. palmitic acid (C16:0) and 0⋅3–1⋅4 g/100 g e.p. stearic acid (C18:0)^(9,14)^. In particular, oleic acid is the most abundant fatty acid (FA) in table olives, and its level differs within the cv. and the processing style. A high proportion of C18:1 was found in brined olives, ranging from 11⋅4 to 36⋅0 g FA/100 g e.p., in green olives from 6⋅9 to 13⋅5 g FA/100 g e.p. and in ripe olives from 5⋅5 to 13⋅8 g FA/100 g e.p.^(9,14)^. Concerning the minor fraction of table olives lipids, the unsaponifiable matter, total sterol contents range from 20 to 30 mg/100 g e.p., although some directly brined olives may have higher concentrations^(14,18)^. The major sterols identified were β-sitosterol, Δ5-avenasterol and campesterol, with overall mean concentrations of 23⋅5, 1⋅5 and 0⋅9 mg/100 e.p., respectively,^(14)^ regardless of the influence of the processing method and cv. The levels of cholesterol in table olives usually are below 0⋅5 mg/100 g e.p., but the stuffing materials from animal or fish origins could increase the total cholesterol content in commercial table olives^(14)^. The mean concentration of total alcohols such as fatty and triterpenic alcohols is 13⋅3 mg/100 g e.p.^(14)^. The main fatty alcohols present in table olives are octacosanol and hexacosanol with average levels of 4⋅7 and 3⋅5 mg/100 e.p., respectively, and the concentrations of triterpenic alcohols are generally even lower than those of fatty alcohols^(14)^ (Table 2).

**Table 1.** Lipid composition of table olives

	Total lipids (g/100 g e.p.)	Saponifiable matter							Unsaponifiable matter (mg/100 g e.p.)		
		SFA (g FA/100 g e.p.)	Palmitic acid (C16:0)	Stearic acid (C18:0)	MUFA (g FA/100 g e.p.)	Oleic acid (C18:1 ω-9)	PUFA (g FA/100 g e.p.)	Linoleic acid (C18:2 ω-6)	Total sterols	Total fatty alcohols	Total triterpenic alcohols
Green olives											
Average*	6–24	2·8	2·3	0·4	9·9	9·6	1·1	1·0			
Gordal cv.*	11·5	–	1·2	–	–	6·9	–	–	26·9	6·7	2·2
Manzanilla cv.*	16·2	–	2·5	–	–	10·7	–	–	27·5	9·1	3·1
Carrasqueña cv.*	13·5	–	–	–	–	–	–	–	25·8	8·5	3·9
Hojiblanca cv.*	14·7	–	–	–	–	–	–	–	29·2	9·3	4·8
Intosso cv.**	17·5	2·7	2·1	0·5	13·6	13·4	1·2	1·1	–	–	–
Bella di Cerignola cv.**	15·5	2·1	1·8	0·3	12·5	12·3	0·9	0·7	–	–	–
Nocellara B. cv.**	19·8	3·9	–	–	13·9	–	2·0	–	–	–	–
Itrana cv.**	17·7	2·8	2·4	0·3	14·0	13·5	0·9	1·0	–	–	–
Ripe olives											
Average*	8–24	2·6	2·2	0·3	10·8	10·6	0·8	0·7	–	–	–
Gordal cv.*	9·2	–	1·2	–	–	5·5	–	–	22·6	4·6	2·6
Manzanilla cv.*	18·2	–	–	–	–	–	–	–	30·1	8·9	3·1
Carrasqueña cv.*	20·6	–	2·9	–	–	13·8	–	–	28·0	7·6	5·5
Hojiblanca cv.*	14·9	–	–	–	–	–	–	–	26·0	6·2	4·2
Cacereña cv.*	15·6	–	–	–	–	–	–	–	20·2	7·6	3·6
Black olives											
Average*	18–28	3·9	3·2	0·5	13·7	13·5	2·3	2·1	–	–	–
Gordal cv.*	19·7	–	–	–	–	–	–	–	32·8	14·3	2·6
Manzanilla cv.*	21·3	–	–	–	–	–	–	–	34·6	22·0	4·3
Hojiblanca cv.*	18·7	–	2·1	–	–	–	–	–	27·8	12·9	2·3
Arbequina cv.*	30·7	–	5·1	–	–	19·2	–	–	54·8	36·0	5·8
Aloreña cv.*	22·0	–	–	–	–	–	–	–	52·6	18·0	7·0
Verdial cv.*	18·9	–	–	–	–	11·4	–	–	33·1	9·2	11·6
Majatica cv.**	46·9	6·3	4·6	1·4	36·7	36·0	4·0	3·8	–	–	–
Taggiasca cv.**	19·9	3·7	–	–	15·2	–	0·9	–	–	–	–
Peranzana cv.**	23·2	4·1	3·4	0·4	17·0	16·3	2·1	2·5	–	–	–
Itrana cv.**	21·7	2·7	2·2	0·4	17·7	17·5	1·3	1·1	–	–	–
Cellina N. cv.**	19·9	4·4	3·9	0·4	14·5	14·1	1·0	0·9	–	–	–

Description of lipidic fraction (saponifiable and unsaponifiable matter) in different types (green, ripe and black olives) and cultivars of table olives·

cv., cultivar; e.p., edible portion; SFA, saturated fat; MUFA, monounsaturated fat; PUFA, polyunsaturated fat.

References: *^(14)^; **^(9)^.

**Table 2.** Sterols and fatty alcohols in table olives

	Unsaponifiable matter					
	Sterols			Fatty alcohols		Triterpenic alcohol
	β-Sitosterol	Δ5-Avenasterol	Campesterol	Octacosanol	Hexacosanol	Erythrodiol
Green olives						
Average***	22·5	1·3	0·9	4·2	3·0	2·3
Bella di Cerignola cv**	89·6	1·5	–	–	–	–
Nocellara B. cv.**	84·5	4·7	–	–	–	–
Itrana cv.**	88·7	4·3	–	–	–	–
Ripe olives						
Average***	20·9	1·3	0·7	3·6	2·4	2·6
Black olives						
Average***	31·8	2·78	1·3	7·5	7·7	4·6
Majatica cv.**	59·1	34·3	–	–	–	–
Peranzana cv.**	77·4	15·9	–	–	–	–
Itrana cv.**	87·8	5·6	–	–	–	–
Cellina N. cv.**	85·4	5·3	–	–	–	–

Quantification of the main sterols, fatty alcohols and triterpenic alcohol in different types and cultivars of table olives.

References: **^(9)^ – values presented in percentage; ***^(21)^ – values presented in mg/100 g e.p.

cv., cultivar; e.p., edible portion.

Table olives are a valuable source of dietary fibre, particularly pectin, hemicelluloses, cellulose and lignin, and the total fibre concentration is of at least 1⋅5 g/100 g e.p., being approximately 3 g/100 g e.p. in the ones already analysed^(12,14)^.

With regard to micronutrients of particular interest in table olives, reported values of α-TOH ranging between 1·3 and 9 mg/100 g e.p. and in 30 samples of table olives analysed for β-carotene values ranged from 0·04 to 0·26 mg/100 g e.p.^(12,22,23)^. The mineral content in table olives is found in the range of 2·0 to 6·9 g/100 g e.p.; in green olives (Gordal, Manzanilla, Hojiblanca and Verdial cvs.), values vary from 4·2 to 5·5 g/100 g e.p., in Hojiblanca ripe olive a value of 2·0 g/100 g e.p. and for Hojiblanca naturally black olives a value of 6·9 g/100 g e.p.^(9,14)^. The fermenting and packing brines used to produce table olives by the Spanish- and Greek-style method of processing determine the high sodium (Na) and NaCl quantity present in some preparations. In Italian table olives, reported values are, respectively, above ≥1·5 and ≥3·75 g/100 g e.p.^(9)^. In fact, the Na and salt values present in 100 g of table olives are close to the recommended maximum level of intake for Na and salt by World Health Organization (WHO), respectively, <2 g Na/d or <5 g NaCl/d^(24)^. By opposition, table olives have a very low level of potassium, contributing with a small proportion to the recommended daily intake for potassium, which is about 3500 mg/d by WHO and European Food Safety Authority (EFSA) guidelines and 4700 mg/d by Food and Nutrition Board of Institute of Medicine (FNB, IOM)^(25–27)^.

## Non-nutritional characteristics of table olives

The main phytochemicals identified and quantified in table olives could be divided into phenolic and non-phenolic compounds, being that phenolic compounds are those present in higher amount. The major phenolic compounds in table olives belong to six different classes, including phenolic alcohols (HT and Ty), flavones (luteolin, luteolin-7-O-glucoside, apigenin and apigenin-7-O-glucoside), flavonols (rutin), anthocyanins (cyanidin-3-O-glucoside), phenolic acids (5-O-caffeoylquinic acid) and a hydroxycinnamic acid derivative (verbascoside)^(12,13,18)^. Triterpenic acids are the main subclass of non-phenolic compounds identified in table olives, notably maslinic and oleanolic acids^(18)^.

Total phenolic compounds have already been analysed and quantified in green, turning colour and black table olives. Among green olives, the reported values vary between 37 mg/100 g (Conservolea cv.)^(28)^ and 150 mg/100 g of flesh (Tonda di Cagliari cv.)^(28–31)^. In turning colour, olives were quantified 171 mg caffeic acid/100 g in Throubes Crete olives^(30)^ and 120 mg/100 g in Manzanilla olives^(32)^. The total phenols in black olives range between 31·65 mg/100 g (Conservolea cv.)^(17)^ and 155 mg caffeic acid/100 g (Kalamon cv.)^(17,28,30)^. According to Table 3, HT is the phenolic compound present in higher amount in table olives, regardless of cv. and method of processing. In green olives, the values of this phenolic alcohol range from 14·8 to 144 mg/100 g of flesh^(28,30)^, in turning colour olives a value of 2 mg/100 g of flesh^(30)^ and in black olives vary from 10 mg/100 g of flesh to 83·3 mg/100 g of fresh weight^(17,28)^. Values for HT among different cvs. are available elsewhere^(17,28–30)^ (Table 3). The phenolic alcohol Ty is present in lower quantity comparatively to HT, since the values already described ranged between 0·4 and 21 mg/100 g of flesh (Table 3)^(10,17,28–30)^. Other phenolic compounds also have been quantified, namely luteolin (0·5–27·5 mg/100 g of flesh^(28)^), luteolin-7-O-glucoside (0·26^(17)^–2·8 mg/100 g of fresh weight^(29)^), verbascoside (1·13–75·6 mg/100 g of dry weight^(10)^), apigenin (2·3 mg/100 g of flesh^(29)^) and rutin (2·05 mg/100 g of dry weight^(10)^ to 4·6 mg/100 g of fresh weight^(17)^) (Table 3). Phenolic acids, such as 5-O-caffeoylquinic acid and protocatechuic acid, are present in small quantities, particularly in brine-processed olives, because they are partly metabolized by the microorganisms during the brining stage^(10,18,30)^.

**Table 3.** Phytochemical composition of table olives

	Green olives					Turning colour olives						Black olives			
	Tsakistes (/of flesh)^(30)^	Azeiteira, Carrasquenha, Redondil and Conserva (/dry weight)^(10)^	Chalkidiki (/of flesh)^(28)^	Conservolea (/of flesh)^(28)^	Tonda di Cagliari (/of fresh pulp)^(29)^	Thrubes Crete (/of flesh)^(30)^	Negrinha de Freixo (/dry weight)^(10)^	Kalamon (/of flesh)^(30)^	Amfissa (/of flesh)^(30)^	Crete (/of flesh)^(30)^	Galega (/dry weight)^(10)^	Negrinha de Freixo (/dry weight)^(10)^	Nychati Kalamata (/of flesh)^(28)^	Conservolea (/of flesh)^(28)^	Thassos (/of flesh)^(28)^
Phenolic compounds (mg/100 g)															
Phenolic alcohols															
HT	114·0	278·4	42·4	14·8	41·0	2·0	182·2	39·0	66·0	21·0	383·3	67·0	25·0–76·0	10·0–34·0	70·5
Ty	21·0	18·0	7·6	2·8	0·4	0·9	21·7	14·0	12·0	6·0	13·9	16·1	9·7	2·8	16·5
Flavones															
Luteolin	–	25·0	0·6	0·5	10·6	–	16·5	–	–	–	16·3	0·96	4·7	2·9	27·5
Luteolin-7-O-glucoside	–	2·6	–	–	2·8	–	n·q·	–	–	–	n·q·	0·8	–	–	–
Apigenin	–	–	–	–	2·3	–	–	–	–	–	–	–	–	–	–
Flavonols															
Rutin	–	–	–	–	–	–	–	–	–	–	2·1	–	–	–	–
Hydroxycinnamic acid derivative															
Verbascoside	–	–	–	–	4·8	–	75·6	–	–	–	47·6	1·1	–	–	–
Non-phenolic compounds (mg/100 g)															
Triterpenic acids															
Maslinic acid	–	–	–	–	–	–	–	–	–	–	–	–	–	–	–
Oleanolic acid	25·0	–	–	–	–	38·0	–	12·0	14·0	25·0	–	–	–	–	–

Quantitative description of the main phenolic and non-phenolic compounds found in different types and cultivars of table olives.

**Table 3.** Phytochemical composition of table olives (continuation)

	Black olives			
	Cellina di Nardò (/fresh weight)^(17)^	Leccino (/fresh weight)^(17)^	Kalamàta (/fresh weight)^(17)^	Conservolea (/fresh weight)^(17)^
Phenolic compounds (mg/100 g)				
Phenolic alcohols				
HT	83·3	55·4	47·7	20·5
Ty	–	–	11·1	–
Flavones				
Luteolin	–	–	–	–
Luteolin-7-O-glucoside	0·9	0·3	–	–
Apigenin	–	–	–	–
Flavolnols				
Rutin	–	–	4·6	4·2
Hydroxycinnamic acid derivative				
Verbascoside	20·8	25·9	1·9	5·3
Non-phenolic compounds (mg/100 g)				
Triterpenic acids				
Maslinic acid	211·7	163·8	195·0	213·3
Oleanolic acid	36·6	54·2	133·3	89·2

n.q., not quantified.

Regarding non-phenolic compounds, especially triterpenic acids, as shown in Table 3, maslinic acid has been quantified in black olives from Cellina di Nardò, Leccino, Kalamàta and Conservolea cvs., with values ranging from 163·8 to 213·4 mg/100 g of fresh weight^(17)^, and oleanolic acid has been analysed in green, turning colour and black olives, with a quantity from 12 to 133·3 mg/100 g of fresh weight^(17,30)^.

## Health effects

To the best of the authors’ knowledge, there are no clinical studies on the literature about the health effects of table olives consumption. In addition, epidemiological data regarding table olives intake and health are not available, either. Therefore, the health effects assigned to table olives were reviewed, assuming that they are primarily related with their FA composition, particularly MUFA, and minor constituents, namely tocopherols and phenolic compounds^(18,33)^.

### Monounsaturated FAs

As previously mentioned, MUFAs are the main lipidic fraction of table olives, being oleic acid the leading FA found in table olives. The Seven Countries Study determined an early interest in the effect of MUFA on cardiovascular disease (CVD), since a low prevalence of coronary heart disease in populations of the Mediterranean region was observed, where diets were rich in olive oil, and as consequence in oleic acid^(34)^. Health effects of dietary fats have been extensively studied and accumulated evidence demonstrates that different types of dietary FA have distinct effects on CVD risk, and the type of fat and its source is more relevant than the total amount of fat of the diet^(35)^.

Most studies, including controlled feeding studies, intended to evaluate the effect of dietary MUFA on CV risk factors, such as blood lipids, blood pressure (BP), glucose metabolism and insulin sensitivity and weight maintenance. It was observed that isocalorically replacement of CHO with MUFA (1 and 5 %) is associated with an increase in high-density lipoprotein cholesterol (HDL-c), a decrease in total cholesterol (TC) and in low-density lipoprotein cholesterol (LDL-c) levels with 1 % of replacement, thus decreasing TC to HDL-c ratio, instead of 5 % of replacement where no significant effect on TC and LDL-c levels was observed^(36–39)^. The replacement of SFA with MUFA was associated with a decrease of LDL-c levels, a slight decrease in HDL-c or a preservation of HDL-c levels, a decrease in TC to HDL-c ratio and a decrease in triacylglycerol (TAG) level in healthy, overweight and obese subjects and those with Diabetes Mellitus 2 (DM-II) and Metabolic Syndrome^(37–41)^. Comparing high MUFA *v*. low MUFA diets, favourable effects of high MUFA diets on TC, LDL-c and in TC to HDL-c ratio were observed^(42)^. Regarding BP, high MUFA diets (>12 %) were associated with a decrease in systolic and diastolic BP in both normotensive and hypertensive subjects, and after 6 months of MUFA-rich diet, a reduction in daily need of anti-hypertensive medication was observed^(40,43–45)^. Dietary MUFAs are not associated with increased risk of developing DM-II in men and women^(46,47)^. In fact, it was observed that a high MUFA diet is associated with a decrease of about 0·21 % in haemoglobin A1c and an improvement in insulin sensitivity^(35,48)^.

Regarding body composition, there was no difference in total body weight and waist circumference among high *v*. low MUFA diets, but a decrease in fat mass (FM) (1·94 kg) was seen with high MUFA diet^(49)^. Later on, it was shown that MUFA consumption is associated with favourable changes in the distribution of FM, with a decrease in central body fat adiposity^(50)^. Additionally, a higher oxidation rate of MUFA, diet-induced thermogenesis and energy expenditure with higher intake of MUFA is reported^(51)^. A high oleic acid content in a meal was shown to elevate post-prandial oleoylethanolamide, an endogenous peroxisome proliferator-activated receptor alpha agonist, that regulates food intake by influencing metabolic and reward systems, leading to better control of appetite sensation, thus reducing energy intake^(52)^.

Since effects on CV risk factors may not directly translate into effects on clinical outcomes of disease, it is important to assess effects of dietary MUFA on CVD morbidity and mortality. Several studies reported an inverse association between MUFA intake and risk of CVD and total mortality^(41,53,54)^. There are, however, some studies with different findings, with non-significant or even positive associations between dietary MUFA intake and CVD morbidity and mortality, that may be influenced by the type of food (animal *v.* plant-sourced) that supplied MUFA^(54)^. In a recent clinical trial, persons at high risk for CV events assigned to an energy-unrestricted MD, supplemented with extra-virgin OO or nuts, had a lower rate of major CV events (myocardial infarction, stroke or death from CV causes) than those with a low fat diet (control group)^(55)^.

Briefly, MUFA-rich diets suggest having a beneficial effect on CVD risk factors, namely blood lipids, BP and parameters of glycaemic control, and on major CV events, though special attention should be given to food source of MUFA, as well as to which macronutrient the results are being compared.

### Vitamin E: α-tocopherol

Vitamin E, the main vitamin present in table olives, is a fat-soluble AO that includes two major classes of biologically active substances: the tocopherols and the related but with less biological activity, tocotrienols^(56,57)^. The most important of these is α-TOH, the isoform preferentially absorbed and maintained in human body, and that presents the highest bioactivity *in vivo*^(56–58)^. The fundamental role of vitamin E is to protect the body from oxidative damage caused by reactive oxygen species (ROS), which are formed through metabolic processes or found in the environment, by scavenging peroxyl radicals^(56,57,59)^. In fact, EFSA approved a health claim about vitamin E related to the protection of body tissues, cells, membranes and lipids from oxidative damage due to its AO activity^(60)^. The presence of α-TOH in cell membranes leads to the protection of unsaturated phospholipids as well as the stabilization of those membranes. Consequently, vitamin E is an important constituent of the cellular AO defence system, which involves enzymatic and non-enzymatic factors, many of which depend on other nutrients^(57)^. In fact, when α-TOH scavenges a peroxyl radical, a tocopheroxyl radical is generated and ascorbic acid (vitamin C) is needed to recycle this newly formed oxidant back to TOH^(61)^.

Notwithstanding the AO functions of vitamin E and its role on inhibition of processes related to the development of atherosclerosis, controlled clinical trials have given changeable results, mostly negative^(57,62)^. α-TOH influences the development of atherosclerosis through decreasing lipid peroxidation, monocyte proatherogenicity and platelet aggregation^(63)^. Furthermore, the potential role of vitamin E in modulating signalling pathways has also been demonstrated, namely those that generate inflammatory molecules, such as inhibition of kinases like 5-lipoxygenase, decreased release of several cytokines and inhibition of NF-κB activation^(63,64)^. Additionally, α-TOH inhibits protein kinase C (PKC)-mediated monocyte superoxide production, smooth muscle cell proliferation and platelet aggregation, and enhances nitric oxide (NO) production^(65–67)^. Despite large observational studies have shown a benefit from vitamin E supplements, controlled clinical trials have produced mixed results. The Nurse's Health Study^(68)^ and Health Professionals Follow-Up Study^(69)^, both observational studies, suggested 20–40 % reductions in heart disease risk among subjects who took vitamin E supplements (generally containing 400 IU or more) for at least 2 years. Among clinical trials there are divergent findings, some with a beneficial effect and others with no benefit found after supplementation with α-TOH in different populations (summarized in Table 4). In brief, those clinical trials studied different populations at the start of the study (from patients with some CV risk factor or CV disease to healthy subjects), different concentrations of α-TOH used in supplements (400–800 IU) and different durations of the supplementation period as well as the clinical endpoints evaluated. Considering several studies, there is no definitive evidence of benefit of α-TOH supplementation for secondary prevention of CV events.

**Table 4.** Clinical trials concerning effects of vitamin E supplementation on CV disease

Study	Study population	Supplementation	Endpoints studied	Results	Reference
CHAOS	Patients with angiographically proven coronary atherosclerosis	400–800 IU/d ~510 d	Cardiovascular death and non-fatal MI as well as non-fatal MI alone	↓ Risk of non-fatal MI	(70)
SPACE	Haemodialysis patients with pre-existing CVD	800 IU/d ~519 d	MI (fatal and non-fatal), ischaemic stroke, peripheral vascular disease (excluding the arteriovenous fistula) and unstable angina; each of the component outcomes, total mortality and CVD mortality	↓ Composite CVD endpoints and MI	(71)
WHS	Healthy women	600 IU/d ~10 years	Composite endpoint of first major cardiovascular event (non-fatal MI, non-fatal stroke or CV death) and total invasive cancer	No overall benefit for major clinical CV events or cancer; no effect on total mortality; ↓ CV mortality ↓ Risk of major cardiac events (≥65 years); ↓ Risk of developing blood clots in the legs and lungs	(72,73)
GISSI	MI survivors	300 mg/d ~3·5 years	Death, non-fatal MI and stroke	No effect on CV outcomes	(74)
HOPE	Adults with high risk of CV events	400 IU/d ~4·5 years	Composite of myocardial infarction, stroke and death from CV causes; unstable angina, congestive heart failure, revascularization or amputation, death from any cause, complications of diabetes and cancer	No effect on CV outcomes; ↑ Risk of heart failure in those who took vitamin E	(75,76)
SU.VI.MAX	Healthy adults	Combination of 120 mg of vitamin C, 30 mg of vitamin E, 6 mg of β-carotene, 100 μg of selenium and 20 mg of zinc ~7·5 years	Incidence of cancer and ischaemic CVD	↓ Risk of cancer and risk of dying from any cause only in men; AO pill did not offer any protection against heart disease	(77)
PHS-II	Healthy men	400 IU/d ~8 years	Major CV events	Vitamin E supplements alone or with vitamin C did not show any protection against heart attacks, strokes or cardiovascular deaths	(78)

IU, International Units; MI, myocardial infarction; CVD, cardiovascular disease; CV, cardiovascular; AO, antioxidant.

Vitamin E is an important nutrient in the development of nervous system and for the protection of peripheral nerves, so it has been hypothesized that vitamin E would also protect the nervous system in ageing^(79)^. Prospective studies showed mixed results, some indicate that vitamin E supplements may be associated with small improvements in cognitive function or lowered risk of Alzheimer's disease and other forms of dementia and lower risk of dying from amyotrophic lateral sclerosis (ALS) compared with individuals who never took vitamin E supplements^(80–84)^. Other studies suggest that obtaining higher intakes of vitamin E from the diet, and not through supplementation, is associated with a reduced risk of Parkinson's disease and in people who already have this disease, the intake of vitamin E supplementation did not slow its progression^(85–87)^. A 3-year randomized controlled trial in individuals with mild cognitive impairment observed that an intake of 2·000 IU of vitamin E daily failed to slow the progression to Alzheimer's disease^(88–90)^. Clinical trials with people who already suffer from ALS have failed to show benefits of vitamin E supplementation, though further research is needed^(91)^.

At the muscular level, it has been shown that α-TOH improves muscle membrane repair and rescues myocytes from necrosis, *in vitro* and in excited mouse muscle studies, thus demonstrating a potential new function of α-TOH in membrane repair^(58)^. Furthermore, α-TOH supplementation may improve muscle function or mitigate functional decline during ageing through the following possible mechanisms: attenuate oxidative stress and suppress inflammation by enhancing AO capacity; improve membrane repair and increase survival of injured skeletal muscle by mitigating oxidized phospholipid formation; improve mitochondrial energetic efficiency; decrease usage of glycogen in skeletal muscle, while increasing fat oxidation; enhance muscle regeneration capacity and stabilize insulin structure and improve insulin sensitivity of skeletal muscle, as supported by animal studies^(92)^.

The role of vitamin E on cancer prevention has shown unpromising results, since observational studies have not found that vitamin E, both in food or supplements, offers great protection against cancer in general or specific cancers^(93–103)^. However, some observational and clinical studies indicated that vitamin E supplements might lower the risk of advanced prostate cancer in smokers^(95,104–106)^.

As previously mentioned, the level of α-TOH in table olives ranges between 1·3 and 9 mg/100 e.p., equivalent to 1·9–13·4 IU on its natural form. The Recommended Dietary Allowance (RDA) of vitamin E as α-TOH is 15 mg to the adult population^(107)^ and the adequate intake for this nutrient is 13 mg to men and 11 mg to women^(108)^. Data from national dietary surveys in European countries show that the estimate adult intakes of vitamin E as α-TOH are higher in men than in women and in the majority of the countries, the estimated average ingestion is lower than the RDA (12·3 mg of α-TOH for men and 10·9 mg for women)^(109–122)^. For this reason, table olives seem a valuable food choice in order to reach the daily need of α-TOH.

### Phenolic compounds

The primordial AO and free radical scavenging activity associated with phenolic compounds present in olives and OO are mainly due to the presence of 3,4-dihydroxy moiety linked to an aromatic ring^(123)^. Once a phenolic compound donates a hydrogen atom to the ROS, it becomes a free radical, but its aromatic ring system stabilizes the newly formed radical, making it virtually non-reactive^(15)^. It is described that the AO activity of phenolic compounds is due its ability to activate some kinases, comprising mitogen-activated protein kinase, PKC and phosphoinositide 3-kinase, thus inducing nuclear factor erythroid 2-related factor 2 (Nrf2) activation^(124,125)^. Nrf2 is a key nuclear transcriptional factor that regulates the expression and activity of several AO and phase II enzymes. Most importantly, the *in vivo* AO effects of phenolic compounds from olives and OO are closely connected to the bioavailability of these compounds from the diet^(126)^. In fact, it was already demonstrated that olive intake leads to an increase in polyphenol levels and AO activities in plasma^(7)^. For instance, it was observed that Tonda di Cagliari cv. phenolic extract significantly counteracted the modification of redox status, inhibiting ROS generation and decrease of glutathione (GSH) level and subsequent membrane oxidative damage,^(29)^ and the daily consumption of 12 green table olives Nocellara del Belice cv. had protective function against oxidative status and inflammatory environment^(127)^.

The anti-carcinogenic activities associated with phenolic compounds are well described both *in vitro* and *in vivo*^(128)^. Phenolic compounds have demonstrated the ability to counteract some essential steps of cancer development, namely apoptosis evasion, angiogenesis, growth signal self-sufficiency, invasion of tissue and insensitivity to antigrowth signals^(129)^.

The best-studied phenolic compounds in olives and OO are OL and HT, but due to the low content of OL in table olives and being the HT, the major phenolic compound found in table olives, the effects of HT will be mainly described. Valuable to note is that bioavailability in human subjects of HT is only proved when it comes from OO and not from table olives, which induces some limitations to extrapolation of the following evidence of HT to a different food matrix.

Health benefits related to HT are mainly attributed to their AO and anti-inflammatory properties^(18)^. The AO effects of HT is due to its ortodiphenolic structure that allows free radical scavenge and radical chain breakdown actions and the formation of metal complexes reducing free radical generation^(130)^. In addition, HT offers an indirect AO protection through activation at gene-level phase II enzymes, including GSH, superoxide dismutase, haeme oxygenase-1 and NAD(P)H quinone oxidoreductase-1, possibly through activation of Nrf2 and AMPK-FoxO3a^(131)^. Once Ty lacks the o-diphenol structure, it has a weak AO activity and it is ineffective in scavenging free radicals compared with HT^(128,132)^.

The anti-inflammatory properties of HT are due to its potential abilities to inhibit NO and prostaglandin E production, decrease secretion of pro-inflammatory cytokines (IL-1, IL-1β, IL-6, IL-12, TNF-α) and chemokines (CXCL10/IP-10, CCL2/MCP-1), decrease gene expression of NO synthase (iNOS), IL-1α, CXCL10/IP-10, CCL4/MIP-1β, matrix metalloproteinase-9 (MMP-9) and PGEs, and modulation of microRNA146a expression and activation of Nrf2^(133–136)^. However, in a clinical trial with intake of 25 mg/d of HT during 1 week, a reduction of plasma CRP and isoprostane levels was observed, but not a reduction on other inflammatory markers such as IL-6, CCL2/MCP-1 and TNF-α^(137)^.

In accordance with the previously mentioned underlying properties of HT, henceforth it will be reviewed some of the most important effects of HT on diseases.

#### Cardiovascular disease

The mechanisms underlying the prevention of atherosclerotic lesion development are limitation of oxidative injury and prevention of LDL-c oxidation, reversion of angiogenesis through the inhibition of MMP-2 and MMP-9 activity, reduction of inflammatory damage induced by inflammatory markers such as TNF-α, decrease of eicosanoid formation and expression of cell adhesion molecules (Vascular Cell Adhesion Molecule1 (VCAM-1)-1 and Intercellular Adhesion Molecule 1 (ICAM-1))^(128,135,138)^. It was observed in human aortic endothelial cells treated with physiological concentrations of HT and co-incubated with TNF-α that a significant reduction of E-selectin, P-selectin, ICAM-1 and VCAM-1 secretion occurred, as well as a decrease of markers of endothelial dysfunction^(139)^. However, some studies observed that in apo-E-deficient mice, the administration of HT enhances atherosclerotic lesion^(140)^. HT can also be considered antithrombotic, since it decreases platelet aggregation, eicosanoid synthesis such as thromboxane B2, leukotriene B4 and to its capacity to reduce cAMP and cGMP platelet phosphodiesterase and to decrease of cell adhesion molecule expression^(138,141)^.

#### Body weight and development of adipose tissue

There is only one clinical study that evaluated the effect of HT supplementation (9·67 mg/d) associated with OL (51·1 mg/d) (for 12 weeks) on body weight and observed that the experiment did not have any effect on this parameter in overweight men^(142)^. The absence of effect on body composition was also proved in rodent models, except for one study that demonstrates the beneficial effect of HT supplementation (50 mg/kg/d × 17 weeks) against diet-induced obesity^(136,143)^. In this experimental study, it was also demonstrated that HT could normalize diabetes, insulin resistance, dyslipidaemia, inflammation and fatty liver induced by high-fat diet^(143)^. It is thought that the mechanisms underlying these effects in obese mice are driven by down-regulation of the sterol regulatory element-binding transcription factor 1c/FA synthase (SREBP-1c/FAS) pathway, while decreasing OS, attenuating mitochondrial abnormalities and suppressing apoptosis^(143)^. In rat hepatocytes, HT down-regulated FA, TG and cholesterol synthesis through decrease of acetyl-CoA carboxylase, diacylglycerol acyltransferase and 3-hydroxy-3-methyl-glutaryl-CoA reductase activities, respectively^(144)^.

#### Cancer

The primary activities of HT linked to the prevention of some types of cancer are inhibition of arachidonic acid metabolism and production of pro-inflammatory compounds, quenching ROS and modulation of proliferation, vascularization and apoptosis of cancer cells by different mechanisms^(15,134,135,138)^. It was observed that HT inhibits the cytotoxicity in human intestinal epithelial Caco-2 and human colon adenocarcinoma HT-29 cancer cell lines through the increment of intracellular capacity to protect against oxidative damage^(145–147)^. Regarding mechanisms underlying modulation of proliferation, a down-regulation of STAT protein family, FAS gene expression, epidermal growth factor receptor and Bcl-2 and COX-2 proteins was already described, through inhibition of p38 transcriptional factor, as well as interference on cell cycle in different cancer cell lines^(15,128,134,138,148)^. On the contrary, treatments of MCF-7 and MDA-MB-231 breast cancer cell lines and MCF10A cell line with physiological concentrations of HT and Ty do not have any effect on cell proliferation or apoptosis, independently of the exposure period, but higher doses of HT were able to decrease the viability of all cell lines studied^(132)^. Inhibition of apoptosis was already described for HT-29 and MCF-7 cancer cell lines, possibly through up-regulation of tumour protein p53^(149–151)^.

Considering that most information about health benefits of HT has been derived from cell culture studies and from animal models, care must be taken with extrapolation of all these findings to human context. Besides this, high concentrations of HT were mainly used in cell studies and in the majority of them, this compound was studied as a single molecule and not with other bioactive compounds that are present in a food matrix, which possibly can influence the bioactivity of HT. However, encouraging results are described and represent a starting point to further animal studies and well-designed controlled trials in humans in order to discover and prove the effects of HT as a sole compound and more importantly, as a part of a food matrix namely table olives.

## Salt reduction in table olives: a potential innovation

A high consumption of sodium (>2 g/d, equivalent to 5 g salt/d) and low consumption of potassium (<3·5 g/d) contribute to high BP and increase risk of CVD and stroke^(152)^. It is suggested that a diet rich in potassium and calcium and poor in sodium prevents the development of osteoporosis and/or colon cancer, in addition to their effects on CV health^(153,154)^. Strategies at individual and governmental levels may be adopted to reduce the intake of NaCl. One of these possible strategies is the reformulation of the products by the food industries, through reduction of final salt content by substitution with other salts, namely potassium chloride (KCl), calcium chloride (CaCl_2_), magnesium chloride (MgCl_2_) and zinc chloride (ZnCl_2_)^(153)^. It is unlikely that one single salt can replace total NaCl and results in a product with acceptable microbiological and organoleptic characteristics for human consumption. For this reason, the effects of replacing some of NaCl for a specific type of food and processing that it underwent must always be studied.

At the microbiological level, the use of CaCl_2_ and ZnCl_2_ usually leads to a decrease of microbiological growth, especially of *Enterobacteriaceae* and yeasts, and the use of KCl is associated with a slight increase in microbiological growth^(153,155)^. The effects of different salts in physicochemical properties of the brine are thought to be: CaCl_2_ – delay of fermentative process by delaying sugar diffusion and consequently in production of lactic acid and decrease of pH, and improved firmness; KCl – slight reduction in production of lactic acid; ZnCl_2_ – delay in sugar consumption and improved firmness^(153,155)^. In contrast, in black table olives it was observed that the partial substitution of NaCl by CaCl_2_ and KCl salts did not change the fermentation, enabling the reduction of overall salt content in the final product and thus, improving its quality^(155)^.

With respect to organoleptic characteristics, it seems that the use of CaCl_2_ influences the firmness, bitterness, hardness, fibrousness and crunchiness, depending on the concentration used^(153)^. The KCl provides similar saltiness to NaCl and the mixture of these salts improves the taste of table olives and likewise the use of ZnCl_2_ improves the overall quality of the final product, mostly by decreasing bitterness^(153,155)^. The simultaneous use of similar proportions of NaCl, KCl and CaCl_2_ salts, in green and black table olives, resulted in a final product with less salt and with acceptable organoleptic characteristics, without any significant influence on end saltiness^(155)^.

Briefly, the applicability of KCl, CaCl_2_ and ZnCl_2_, as partial substitutes for NaCl, in table olives seems promising since they do not appear to significantly alter the microbiological and organoleptic characteristics of the final product.

## Conclusion

Table olives have an important nutritional value due to its richness in MUFA, fibre, vitamin E and phenolic compounds. The high amount of salt that may be present in the final product represents a negative factor in the overall nutritional quality of the end product. Even though the consumption of table olives occurs occasionally and in moderate amounts, efforts should be made to reformulate this product in order to decrease its salt content. The possible health benefits associated with consumption of table olives are primarily related to effects of MUFA on CV risk factors and on major CV events, the role of vitamin E in protection of the body from oxidative damage and the AO and anti-inflammatory properties of HT. Most information has been driven from cell culture studies and from animal studies. The need for further research, namely well-designed human studies, is imperative to determine the conditions under which table olives will provide health benefits.

Taken together, the consumption of table olives in a moderate amount should be encouraged in the context of a healthy dietary pattern, as a snack or appetizer.
